# Study on the Constitutive Model for Jointed Rock Mass

**DOI:** 10.1371/journal.pone.0121850

**Published:** 2015-04-17

**Authors:** Qiang Xu, Jianyun Chen, Jing Li, Chunfeng Zhao, Chenyang Yuan

**Affiliations:** 1 State Key Lab.of Coastal and Offshore Eng., Dalian University of Technology, Dalian, China; 2 School of Civil and Hydraulic Eng., Dalian University of Technology, Dalian, China; University of Vigo, SPAIN

## Abstract

A new elasto-plastic constitutive model for jointed rock mass, which can consider the persistence ratio in different visual angle and anisotropic increase of plastic strain, is proposed. The proposed the yield strength criterion, which is anisotropic, is not only related to friction angle and cohesion of jointed rock masses at the visual angle but also related to the intersection angle between the visual angle and the directions of the principal stresses. Some numerical examples are given to analyze and verify the proposed constitutive model. The results show the proposed constitutive model has high precision to calculate displacement, stress and plastic strain and can be applied in engineering analysis.

## Introduction

In the rock engineering, joints have significant effect on the stress-strain relationship of jointed rock mass. Generally speaking, there are two categories of approaches: the first method is that joint element is utilized to simulate jointed rock mass. The other method is that special constitutive model is utilized to simulate jointed rock mass.

Constitutive models for jointed rock masses are important for numerical modeling of the behavior of jointed rocks. Many constitutive models for rock joints, based on both empirical and theoretical approach, such as are summarized in [1]. The behavior of the joints is dependent on their sizes, because the scale dependence of surface roughness of the joints whose thresholds are a scaling parameter [2–4]. Some researchers took study on landslide problems and the dynamic frictional processes of the joints using theories of dynamic chaos and catastrophe for an analysis of the interactions between the fracture surfaces regarding friction, fracture stiffness and elastic materials for the jointed rocks[5]. Some researchers utilized joint factor to simulate jointed rock mass based on the finite element method[6]. Some researchers proposed the model for the equivalent elastic parameters of jointed rock mass[7,8]. Some researchers performed the modeling of dynamic rock fracture sliding using the state variable friction models. In the model, the shear stresses are the functions of both the sliding history and velocity. And the model represented the evolution of rate effects and the path-dependence of the frictional properties[9]. Some researchers utilized representative volume method to analyze nonlinear characteristics of one-way joint and the interaction of two-way orthogonal joints[10]. Some researchers reported new 3D constitutive models for rough rock fractures based on experimentally determined relations between the contact areas under normal loads and asperity inclination angles[11,12]. Some researchers established the model to calculate the physical parameters of jointed rock mass[13]. Some researchers established softening model for multi-joints[14].

In this paper, the studies on elasto-plastic constitutive model for jointed rock mass are made. The influences of joints on the jointed rock mass are analyzed. Based on these studies, a constitutive model for jointed rock mass, which can consider anisotropic strength of jointed rock mass and anisotropic increase of plastic strain, is constructed. And then the numerical examples are performed to analyze and verify the proposed constitutive model.

## The Constitutive Model for Jointed Rock Mass

### 2.1 The construction of constitutive model

Morh-Coulomb model is well-known model in geotechnical engineering application, including in rock engineering modelling and design. The basic concepts of the Mohr-Coulomb model suggest that the behaviors of a rock material are made up of two parts: a constant cohesion and a friction coefficient. And it can be described as

$$\tau_{s}=\sigma_{n}tg\varphi \text{+}c$$ (1)

where *τ*_s_ is the shear strength, *σ*_n_ is the normal stress, *c* is the cohesion, *φ* is friction angle. The parameters of this model are only two, and it is widely used due to the simple expression. But this model is based on the isotropy theory. It can only describe the isotropic material. And jointed rock mass is anisotropic material. The classical Morh-Coulomb model cannot describe the behaviors of jointed rock. So it need to be improved due to its limitations.

**Fig 1:**
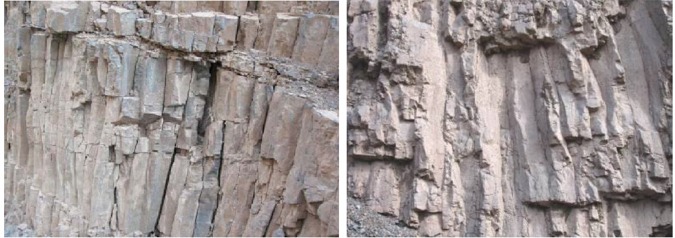
The joint net work of rocks

**Fig 2:**
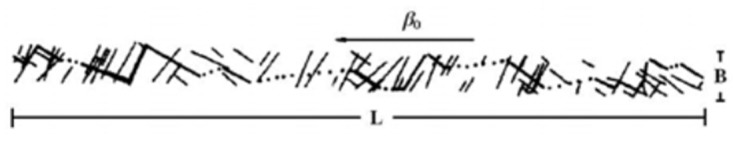
The failure path of joints and rock bridges

Fig 1 shows rock bridges exist in jointed rock masses because of the non-persistent nature of joints. In order to calculate the decrease of strength of jointed rock masses in different directions, It defines the mechanical persistence ratio of rock mass as that the ratio of joint network on the shear failure path when jointed rock mass is sheared to damaged state along a certain direction[15]. Fig 2 shows that the mechanical persistence ratio *k* is calculated by

$$k_{\beta_{0}}=\frac{\sum JL}{\sum JL+\sum RBR}$$ (2)

where *JL* and *RBR* are the projection length of joints and rock bridges in the shear failure path respectively. *β*_0_ is the visual angle, which can be used to express the direction of joints.

It defines cohesion *c*_*β*0_ and friction angle *φ*_*β*0_ of jointed rock masses in direction of *β*_0_ [16,17]as

$$c_{\beta_{0}}=(1-k)c_{r}+kc_{j}$$ (3)

$$tan\varphi_{\beta_{0}}=(1-k)tan\varphi_{r}+ktan\varphi_{j}$$ (4)

where *c*_r_ and *φ*_r_ are the cohesion and friction angle of rock bridges, respectively. *c*_i_ and *φ*_i_ are the cohesion and friction angle of joints.

Thus, based on Mohr-Coulomb model, the yield strength criterion *f* can be given by

$$f=|\tau |+\sigma tg\varphi_{\beta_{0}}-c_{\beta_{0}}$$ (5)

where *τ* and *σ* are the shear stress and normal stress in direction of *β*_0_, respectively.

**Fig 3:**
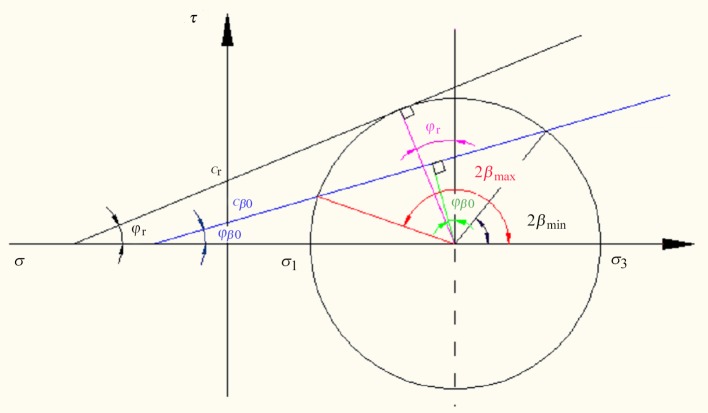
Mohr circle of stress for jointed rock masses

The Mohr-Coulomb model is based on plotting Mohr's circle for states of stress at failure in the plane of the maximum and minimum principal stresses. According to Fig 3, we have

$$\sigma =\frac{1}{2}(\sigma_{3}+\sigma_{1})+\frac{1}{2}(\sigma_{3}-\sigma_{1})cos2\beta$$ (6)

$$|\tau |=\frac{1}{2}(\sigma_{1}-\sigma_{3})sin2\beta$$ (7)

where *σ*_1_ and *σ*_3_ are the maximum and minimum principal stresses, respectively. *β* is the intersection angle between *β*_0_ and the directions of the maximum principal stresses *σ*_1_.

Thus, the yield strength criterion *f* in plane can be rewritten as

$$f=\{\begin{array}{c} \begin{array}{l} \frac{1}{2}\left(\sigma_{1}-\sigma_{3}\right)\sin2\beta +\left(\frac{1}{2}\left(\sigma_{3}+\sigma_{1}\right)+\frac{1}{2}\left(\sigma_{3}-\sigma_{1}\right)\cos2\beta \right)tg\varphi_{\beta_{0}}-c_{\beta_{0}}\\ ,when\;\beta_{\min}\leq \beta \leq \beta_{\max} \end{array}\\ \begin{array}{l} \frac{1}{2}\left(\sigma_{1}-\sigma_{3}\right)\cos\varphi_{r}+\left(\frac{1}{2}\left(\sigma_{3}+\sigma_{1}\right)+\frac{1}{2}\left(\sigma_{3}-\sigma_{1}\right)\sin\varphi_{r}\right)tg\varphi_{r}-c_{r}\\ ,when\;\beta <\beta_{\min}\;\;or\;\;\beta >\beta_{\max} \end{array} \end{array}$$ (8)

in which

$$2\beta_{\min}=\varphi_{\beta_{0}}+\sin^{-1}\left(\left(1+\frac{\left(c_{\beta_{0}}ctg\varphi_{\beta_{0}}-\sigma_{1}\right)\left(1-\sin\varphi_{r}\right)}{-\sigma_{1}\sin\varphi_{r}+c_{r}\cos\varphi_{r}}\right)\sin\varphi_{\beta_{0}}\right)$$ (9)

$$2\beta_{max}=\pi +2\varphi_{\beta_{0}}-2\beta_{min}$$ (10)

From (8), through calculating d*f*/d*β* = 0, we can obtain the least angle *β*_L_ in *β* when *β*_min_≤*β*≤*β*_max_ and we have

$$\beta_{L}=45^{{}^{\circ}}+\frac{\varphi_{\beta_{0}}}{2}$$ (11)

And the minimum value of *σ*_1_ and *σ*_3_ obey the function *f*_min_ when *β* = *β*_L_, and we have

fmin=12(σ1,min-σ3,min)cosφβ0+(12(σ3,min+σ1,min)+12(σ3,min-σ1,min)sinφβ0)tgφβ0-cβ0 (12)

According to Fig 3, we also have

$$\sigma_{m}=\frac{1}{2}(\sigma_{1}+\sigma_{3}),\tau_{m}=\frac{1}{2}(\sigma_{1}-\sigma_{3})$$ (13)

where *σ*_m_ and *τ*_m_ are the mean normal stress and the maximum shear stress, respectively.

Thus, the yield strength criterion *f* in plane can be rewritten as

$$f=\{\begin{array}{c} \tau_{m}\left(\sin2\beta +tg\varphi_{\beta_{0}}\cos2\beta \right)+\sigma_{m}tg\varphi_{\beta_{0}}-c_{\beta_{0}},\;when\;\;\beta_{\min}\leq \beta \leq \beta_{\max}\\ \tau_{m}\sec\varphi_{r}+\sigma_{m}tg\varphi_{r}-c_{r},when\;\beta <\beta_{\min}\;\;or\;\;\beta >\beta_{\max} \end{array}$$ (14)

The yield strength criterion *f* in plane is extended to three-dimensional yield strength criterion and we have

$$f=\{\begin{array}{c} R_{mc,\beta_{0}}q-p\tan\varphi_{\beta_{0}}-c_{\beta_{0}},\;when\;\;\beta_{\min}\leq \beta \leq \beta_{\max}\\ R_{mc,r}q-p\tan\varphi_{r}-c_{r},\;when\;\;\beta <\beta_{\min}\;or\;\beta >\beta_{\max} \end{array}$$ (15)

in which

$$R_{mc,\beta_{0}}=\frac{1}{\sqrt{3}cos\varphi_{\beta_{0}}}sin(\theta +\frac{\pi }{3})+\frac{1}{3}cos(\theta +\frac{\pi }{3})tan\varphi_{\beta_{0}}$$ (16)

$$R_{mc,r}=\frac{1}{\sqrt{3}cos\varphi_{r}}sin(\theta +\frac{\pi }{3})+\frac{1}{3}cos(\theta +\frac{\pi }{3})tan\varphi_{r}$$ (17)

$$p=I_{1}/3,q=\sqrt{3}\sqrt{J_{2}},cos(3\theta )={\left(J_{3}/q\right)}^{3}$$ (18)

where *I*_1_, *J*_2_ and *J*_3_ are the first invariant of stress tensor, the second and third invariant of deviatoric stress tensor, respectively.

In plasticity theory, the strain increment can be decomposed into two parts

$${d\varepsilon =d\varepsilon }^{e}+{d\varepsilon }^{p}$$ (19)

where **d*ε*** is the incremental strain tensor; **d*ε*** ^e^ and **d*ε*** ^p^ are the incremental elastic and plastic strain tensor, respectively.

The stress—strain relationship is expressed as

$$d\sigma^{\prime }=D^{ep}:d\varepsilon$$ (20)

where **d*σ***’ is the incremental stress tensor; ***D*** ^ep^ is the elasto-plastic stiffness tensor.

The elasto-plastic stiffness tensor is expressed as:

$$D^{ep}=D^{e}-\frac{D^{e}:n_{g}:n^{T}:D^{e}}{n^{T}:D^{e}:n_{g}}$$ (21)

in which

$$n_{g}=\frac{\partial g}{\partial \sigma },n=\frac{\partial f}{\partial \sigma }$$ (22)

where ***σ*** is the stress tensor; ***D*** ^e^ is the elasto stiffness tensor; ***n*** and ***n***_g_ are the loading and flow direction vectors, respectively; *f* and *g* are the yield and plastic potential functions, respectively. And in the model, the plastic potential function *g* is adopted as the same as the yield function *f*.

The fluidity variable *Λ* can be expressed as

$$\Lambda =\frac{n^{T}:D^{e}:d\varepsilon }{n^{T}:D^{e}:n_{g}}$$ (23)

The distinction between loading and unloading directions is described through the following criteria:

Λ>0 (loading)  Λ≤0 (unloading) (24)

Because the plastic strain will also increase in the process of reloading, the incremental plastic strain is

$${d\varepsilon }^{p}=\langle \Lambda \rangle n_{g}$$ (25)

where the symbol〈〉is defined as〈*Λ*〉 = *Λ* for *Λ*>0 and〈*Λ*〉 = 0 for *Λ*≤0. It shows that the plastic strain will increase if jointed rock mass is in the state of loading. In other word, we have

$$\left\{ \begin{array}{l} {d\varepsilon }^{p}\neq \text{0, in the plastic state when }\Lambda >0(\text{loading})\\ {d\varepsilon }^{p}\text{=0, in the elastic state when }\Lambda \leq 0(\text{unloading}) \end{array}\right.$$ (26)

### 2.2 Numerical implementation

**Fig 4:**
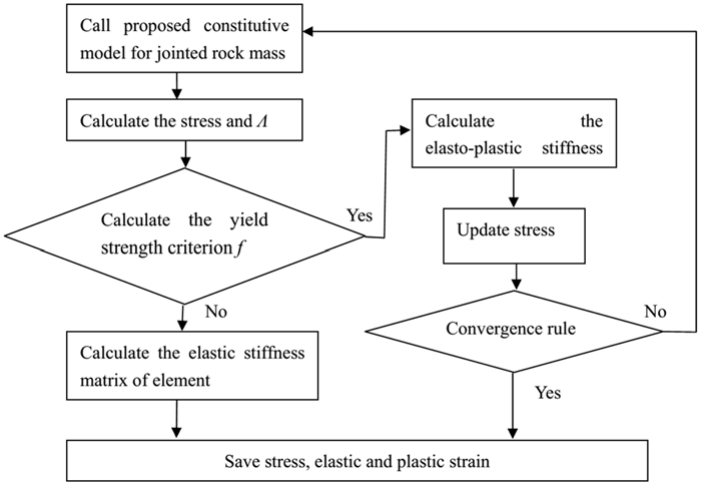
The flow chart of iterative steps of proposed constitutive model

The integral algorithm based on fully implicit backward Euler return mapping algorithm is adopted to calculate the updated stresses. The convergence rule is adopted according to the difference of updated stresses less than tolerance. Fig 4 shows the iterative steps of proposed constitutive model.

## Numerical Examples and Results

### 3.1 The numerical example for the strength of jointed rock mass

**Fig 5:**
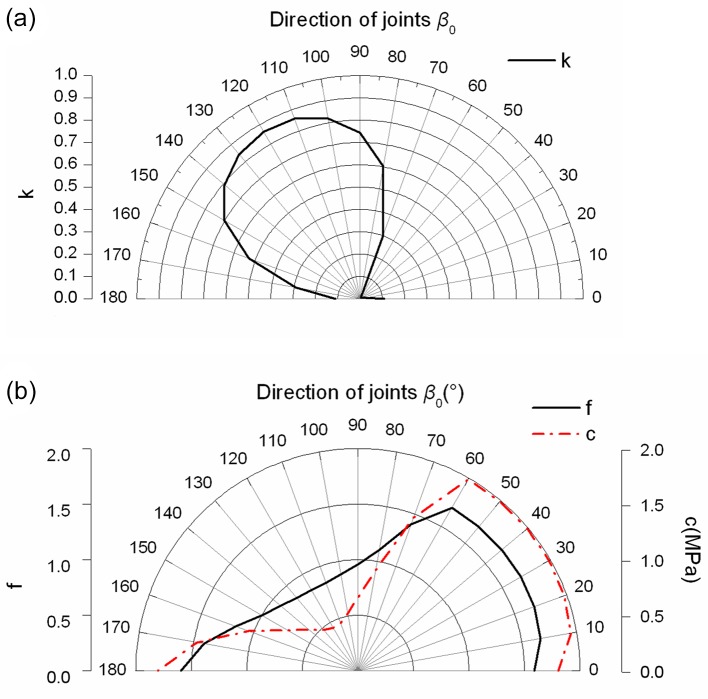
The rose diagrams of the persistence ratio, friction coefficient and cohesion((a) The rose diagrams of the persistence ratio of jointed rock mass; (b) The rose diagrams of friction coefficient and cohesion of jointed rock mass)

In order to analyze the strength of jointed rock mass calculated by proposed elasto-plastic constitutive model, the tests for numerical simulating jointed rock mass are taken. The elastic modulus *E* and Poisson ratio *v* of jointed rock mass are 4.00GPa and 0.25, respectively. Table 1 shows the strength parameters of the joint surface and the rock bridge. Fig 5 shows the persistence ratio, friction coefficient and cohesion of jointed rock mass at the visual angle *β*_0_.

**Table 1:** The strength parameters of the joint surface and the rock bridge

Material	Friction coefficient *f*	Cohesion *c*(MPa)
Joints	0.7	0.2
Rock	1.7	2.0

**Fig 6:**
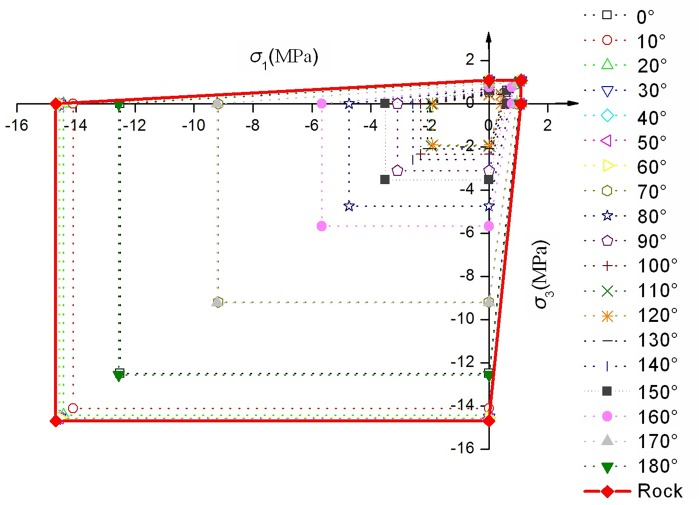
The relation between strength in *σ*_1_-*σ*_3_ plane and the visual angle *β*_0_

**Fig 7:**
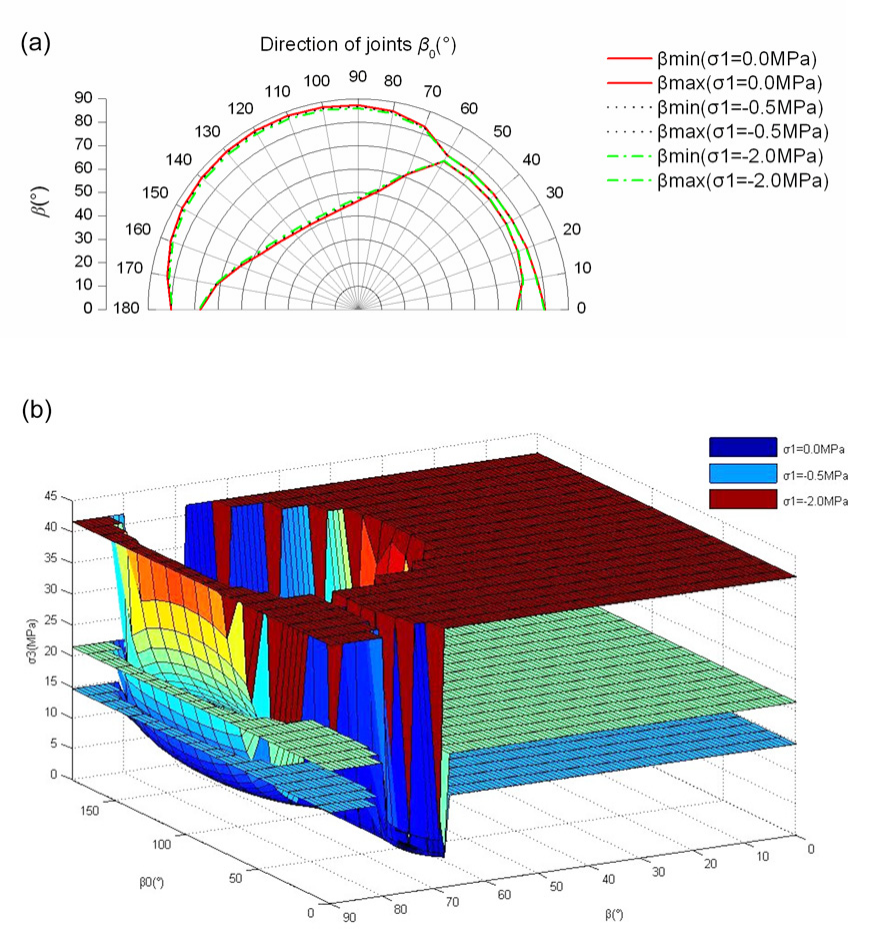
The strength of *σ*_3_ when *σ*_1_ is given((a) The change of *β*_min_ and *β*_max_ with different visual angle *β*_0_; (b) The change of strength of *σ*_3_ with different *β*_0_ and *β*)

**Fig 8:**
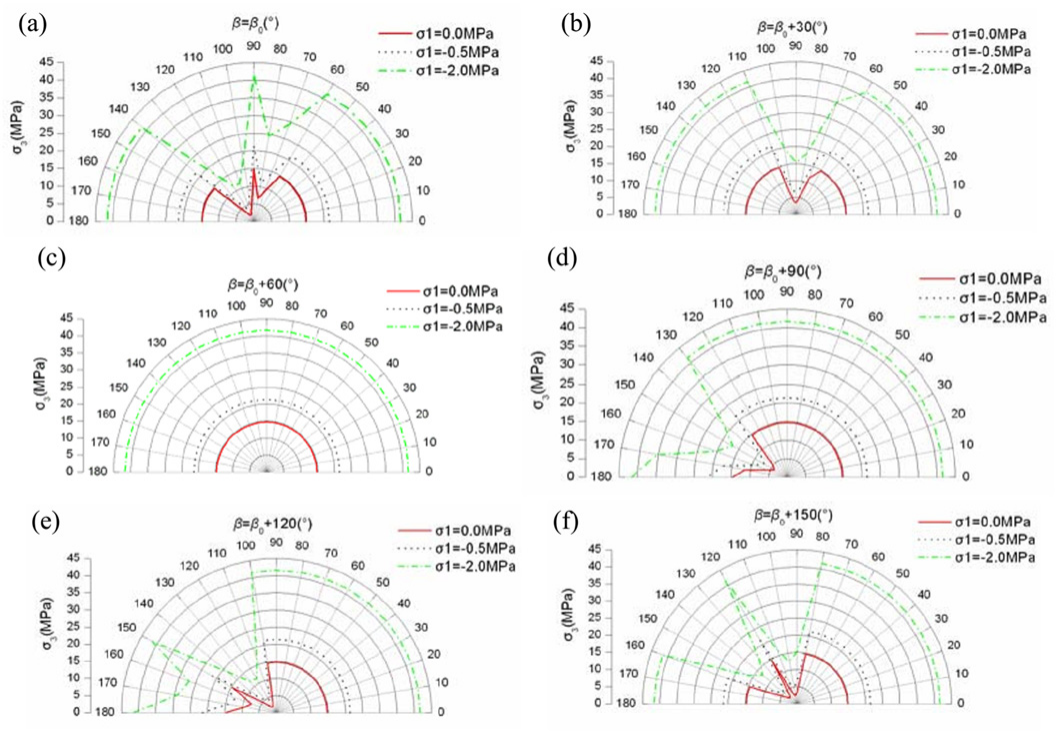
The strength of *σ*_3_ when the relation of *β*_0_ and *β* is given((a) The strength of *σ*_3_ when *β* = *β*_0_; (b) The strength of *σ*_3_ when *β* = *β*_0_+30°; (c) The strength of *σ*_3_ when *β* = *β*_0_+60°; (d) The strength of *σ*_3_ when *β* = *β*_0_+90°; (e) The strength of *σ*_3_ when *β* = *β*_0_+120°; (f) The strength of *σ*_3_ when *β* = *β*_0_+150°)

Through observing the results of Figs 5–8, they show that the yield strength criterion *f* in plane of jointed rock mass is not only related to the friction angle *φ*_*β*0_ and cohesion *c*_*β*0_ of jointed rock masses in direction of *β*_0_(the visual angle). The yield strength criterion *f* is also related to *β* (the intersection angle between the visual angle and the directions of the maximum principal stresses). The yield strength criterion *f* has the relation of *φ*_*β*0_ and *c*_*β*0_ only when *β*_min_≤*β*≤*β*_max_. The relation of *β* and *β*_0_ is also important to the yield strength criterion *f*. The different relation of *β* and *β*_0_ leads to different yield strength criterion *f*. In some special relation of *β* and *β*_0_, such as Fig 8 (c), the friction angle *φ*_*β*0_ and cohesion *c*_*β*0_ has no use for the yield strength criterion *f*. In other word, the persistence ratio *k* has no use for the yield strength criterion *f* in some special condition.

### 3.2 Jointed rock direct shear experiment and numerical simulation by proposed model

**Fig 9:**
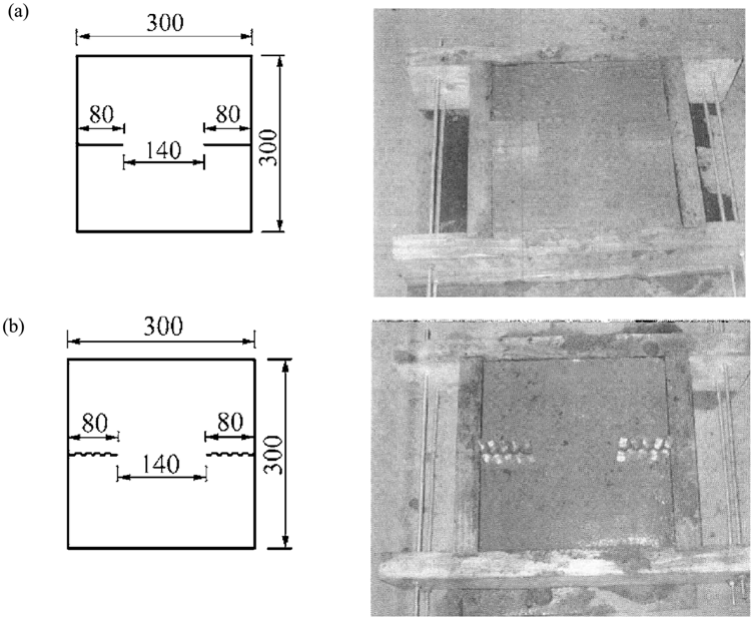
The rock mass samples containing joints[18] ((a)The first rock mass sample containing joints (the visual angles *β*_0_ of joints = 0°);(b)The second rock mass sample containing joints (the visual angles *β*_0_ of joints = 30°))

**Fig 10:**
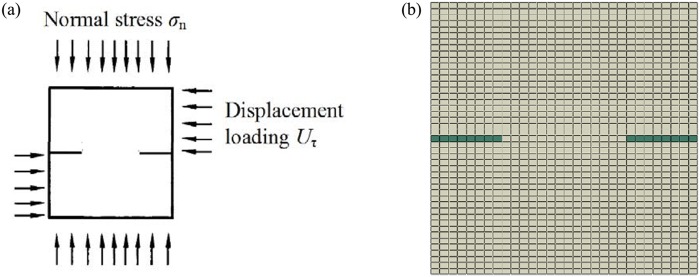
The boundary conditions and numerical model((a)The boundary conditions of jointed rock direct shear experiment;(b) The numerical model calculated by proposed constitutive model)

To verify proposed constitutive model, the compared results of proposed model and experiment are given. The rock mass samples containing joints are 0.3m×0.3m. The visual angles *β*_0_ of joints of two rock mass samples are 0°and 30°,respectively. And the positions of joints are shown in Fig 9. The boundary conditions and numerical model are shown in Fig 10. The normal stress is 1.0MPa, and the shear displacements of experiment are taken to 5mm. The parameters of the joints and the rock mass samples are shown in Table 2.

**Table 2:** The parameters of the joints and the rock

Material	The elastic modulus (GPa)	Poisson ratio	Friction coefficient *f*	Cohesion *c*(MPa)
Joints	0.50	0.16	0.25	0.10
Rock	3.70	0.16	0.89	3.93

**Fig 11:**
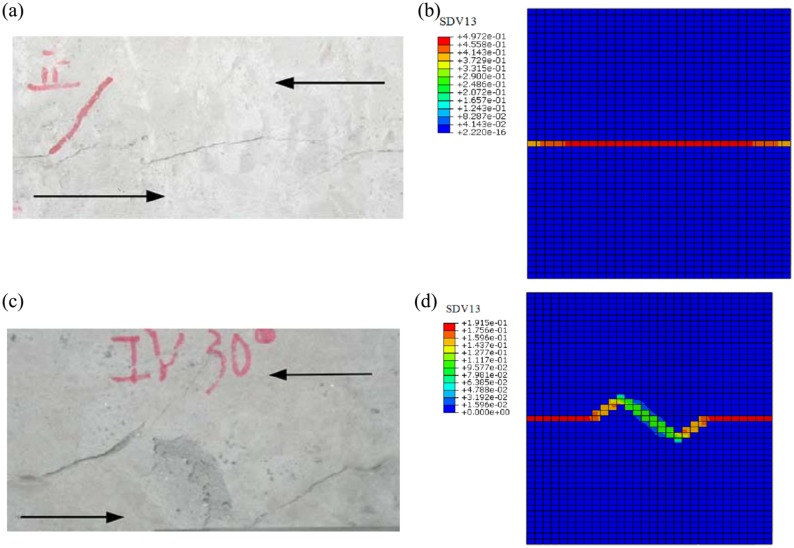
The results of experiment and numerical simulation((a) The failure mode of first rock mass sample in jointed rock direct shear experiment (the visual angles *β*_0_ of joints = 0°);(b) The contour for equivalent plastic deviator strain calculated by proposed constitutive model(the visual angles *β*_0_ of joints = 0°);(c) The failure mode of of second rock mass sample in jointed rock direct shear experiment (the visual angles *β*_0_ of joints = 30°); (d)The contour for equivalent plastic deviator strain calculated by proposed constitutive model(the visual angles *β*_0_ of joints = 30°))

**Fig 12:**
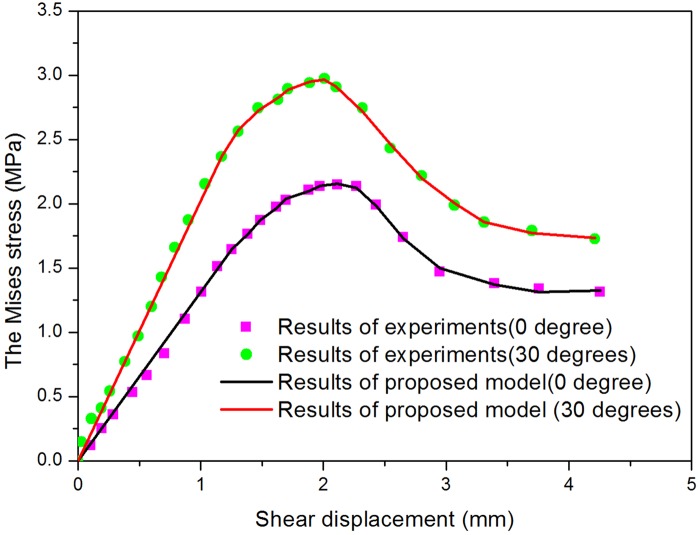
Curves of shear stress-displacement of experiment and numerical simulation

Through observing the results of Fig 11 and Fig 12, they show that the results of failure mode of rock mass by experiment and numerical simulation by proposed model are similar. And the curves of shear stress-displacement of experiment and numerical simulation by proposed model are close. These results verify the proposed constitutive model. And it shows proposed model can describe the behavior of jointed rock well.

### 3.3 The numerical examples for a rectangle foundation of jointed rock mass

**Fig 13:**
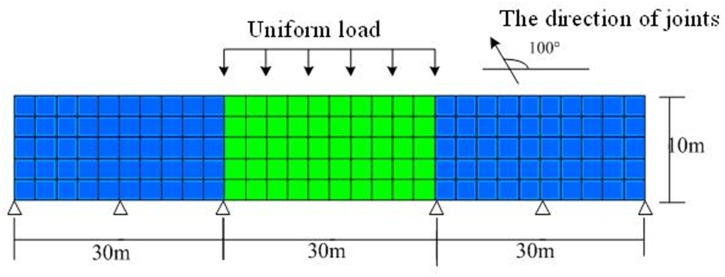
The model for FE analyses with difference materials

Fig 13 shows the plane stress model (finite element(FE) with 4-nodes)for a rectangle foundation of jointed rock mass (length = 120m, depth = 10m) subjected to uniform load *p* = 2GPa. The visual angle *β*_0_ = 100°. There are two kinds of materials in the rectangle foundation. The blue regions are calculated by linear elastic constitutive model. The green region is calculated by proposed constitutive model and ubiquitous-joint constitutive model[19] in commercial software Abaqus, respectively. Table 3 shows the physical parameters of the rectangle foundation.

**Table 3:** The parameters of the rectangle foundation

	Elastic modulus(GPa)	Poisson ratio	Friction angle(°)	Cohesion(MPa)
Linear elastic material	25	0.3	-	-
The parameters of jointed rock mass in the visual angle *β*_0_	25	0.3	35	0.27

**Fig 14:**
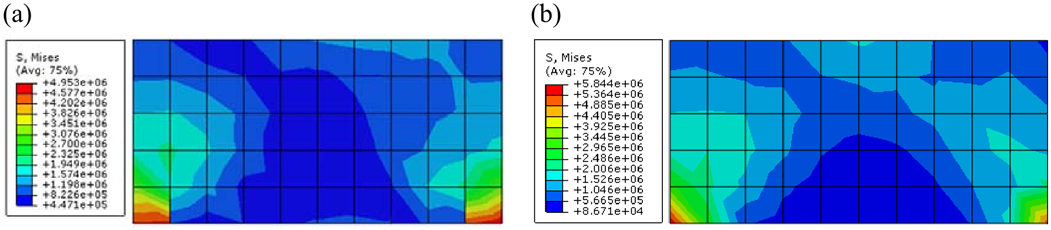
The Mises stress contour of the green region of the rectangle foundation calculated by different constitutive models(Pa) ((a)The Mises stress contour calculated by proposed constitutive model; (b)The Mises stress contour calculated by ubiquitous-joint model)

**Fig 15:**
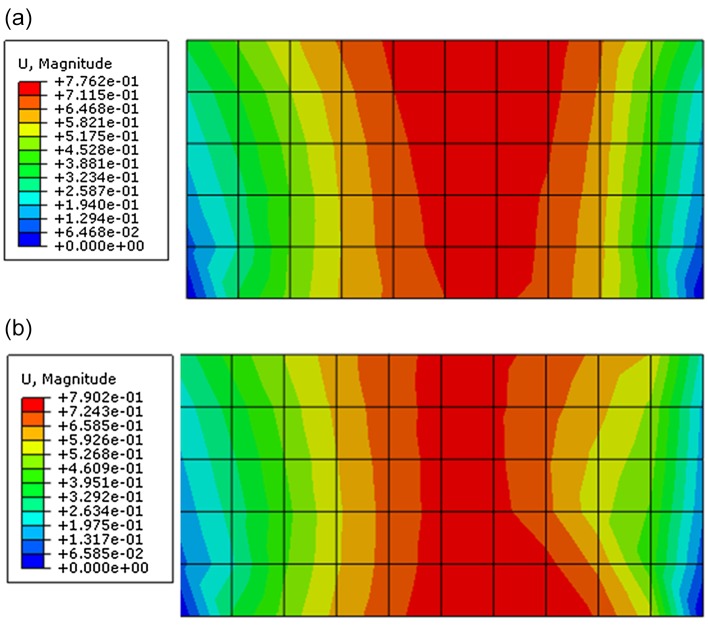
The displacement contour of the green region of the rectangle foundation calculated by different constitutive models(m) ((a)The contour for magnitude of displacement calculated by proposed constitutive model; (b) The contour for magnitude of displacement contour calculated by ubiquitous-joint model)

Through observing the results of Fig 14 and Fig 15, they show that the results of displacement and stress calculated by proposed constitutive model are close to that calculated by ubiquitous-joint constitutive model in commercial software Abaqus, which has been verified. The maximum relative errors of results of displacement and Mises stress calculated by proposed model are 1.77% and 15.25%, respectively.

### 3.4 The numerical example for a rectangle beam of jointed rock mass

**Fig 16:**
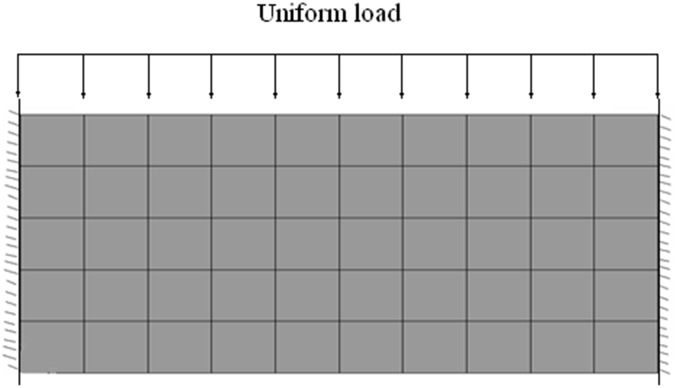
The FE model of the beam

Fig 16 shows the plane strain model(FE with 4-nodes) for a rectangle beam (length = 4m, width = 2m) of jointed rock mass subjected to uniform load *p* = 0.45MPa. Both sides of beam are restraint against displacement. The visual angle *β*_0_ = 120°. The beam is calculated by proposed constitutive model and ubiquitous-joint constitutive model in commercial software Abaqus, respectively. Table 4 shows the physical parameters of the rectangle beam.

**Table 4:** The parameters of the rectangle beam

	Elastic modulus(GPa)	Poisson ratio	Friction coefficient *f*	Cohesion(MPa)
Rock	25	0.3	1.0	1.18
The parameters of jointed rock mass in the visual angle *β*_0_	25	0.3	0.74	0.33

**Fig 17:**
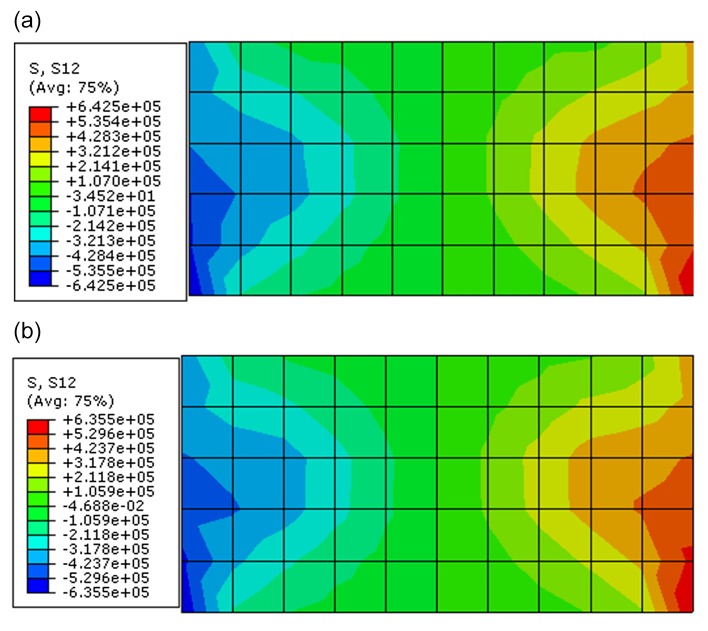
The shear stress contour of the rectangle beam calculated by different constitutive models(Pa) ((a) The shear stress contour calculated by proposed constitutive model; (b) The shear stress contour calculated by ubiquitous-joint model)

**Fig 18:**
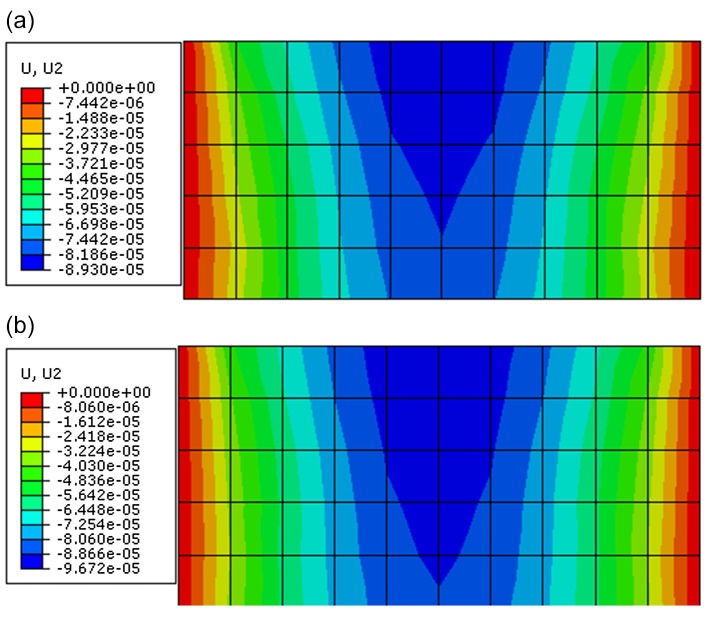
The vertical displacement contour of the rectangle beam calculated by different constitutive models(m) ((a) The vertical displacement contour calculated by proposed constitutive model; (b) The vertical displacement contour calculated by ubiquitous-joint model)

**Fig 19:**
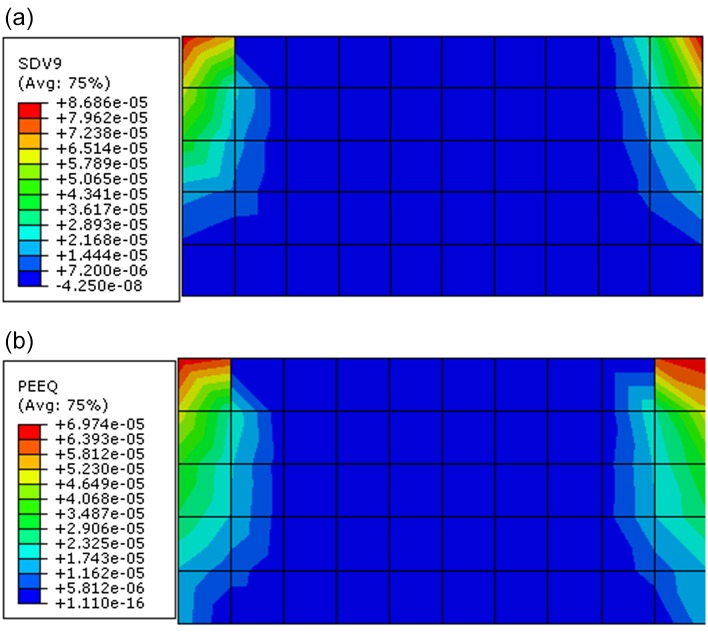
The contour for equivalent plastic deviator strain of the rectangle beam calculated by different constitutive models((a) The contour for equivalent plastic deviator strain calculated by proposed constitutive model;(b) The contour for equivalent plastic deviator strain calculated by ubiquitous-joint model)

Through observing the results of Figs 17–19, they show that the results of displacement, stress and plastic strain calculated by proposed constitutive model are also close to that calculated by ubiquitous-joint constitutive model in commercial software Abaqus. The maximum relative errors of results of displacement, stress and plastic strain calculated by proposed model are 8.31%, 1.08% and 19.71%, respectively. The results show the proposed constitutive model has some precision and verify the proposed constitutive model.

### 3.5 The numerical example for slope

**Fig 20:**
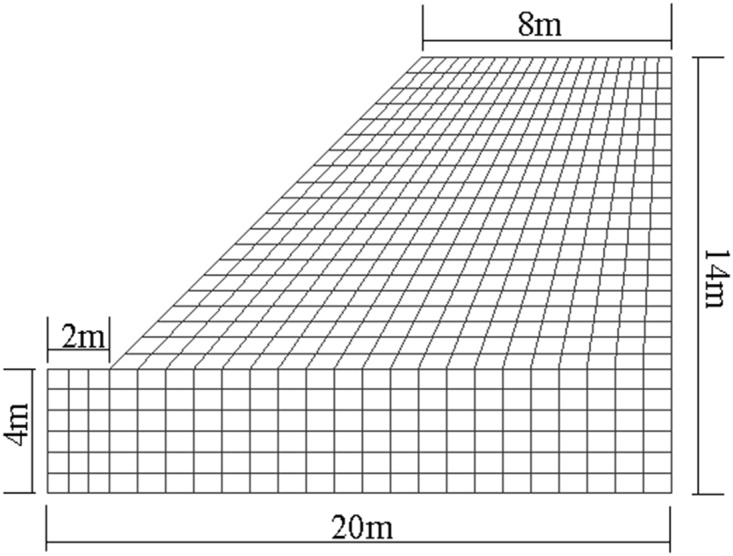
The model of non-homogeneous rock slope

**Fig 21:**
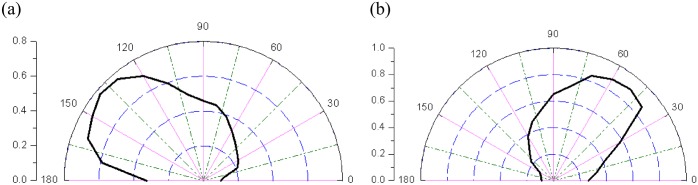
The rose diagrams of the persistence ratio *k* of jointed rock mass for the different slopes((a) The rose diagrams of the persistence ratio *k* of jointed rock mass for the first slope; (b) The rose diagrams of the persistence ratio *k* of jointed rock mass for the second slope)

Fig 20 shows the plane stress model (FE with 4-nodes) for a slope of jointed rock mass subjected to gravity. The elastic modulus and Poisson ratio of jointed rock mass are 4.00GPa and 0.30, respectively. Fig 21 shows two kinds of jointed rock mass, whose persistence ratios are different, are used to calculate. Table 5 shows the parameters of slope.

**Table 5:** The parameters of slope

Material	Friction coefficient *f*	Cohesion *c*(MPa)
Joints	0.2	0.1
Rock	1.9	1.1

**Fig 22:**
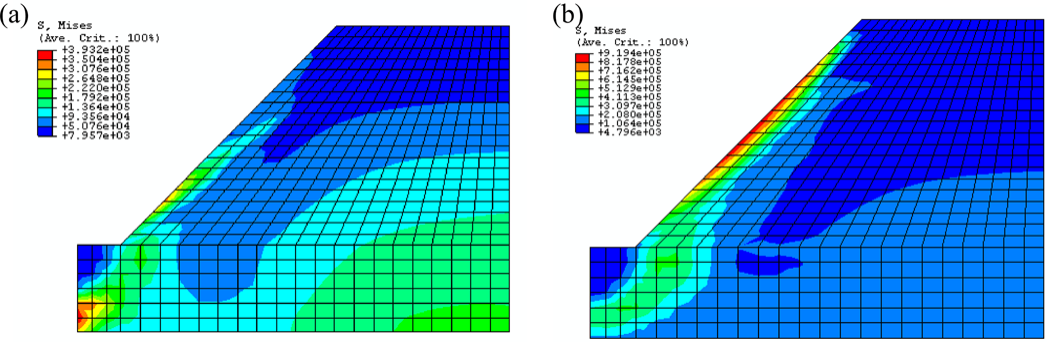
The Mises stress contour calculated by proposed constitutive model for the different slopes((a) The Mises stress contour calculated by proposed constitutive model for the first slope; (b) The Mises stress contour calculated by proposed constitutive model for the second slope)

**Fig 23:**
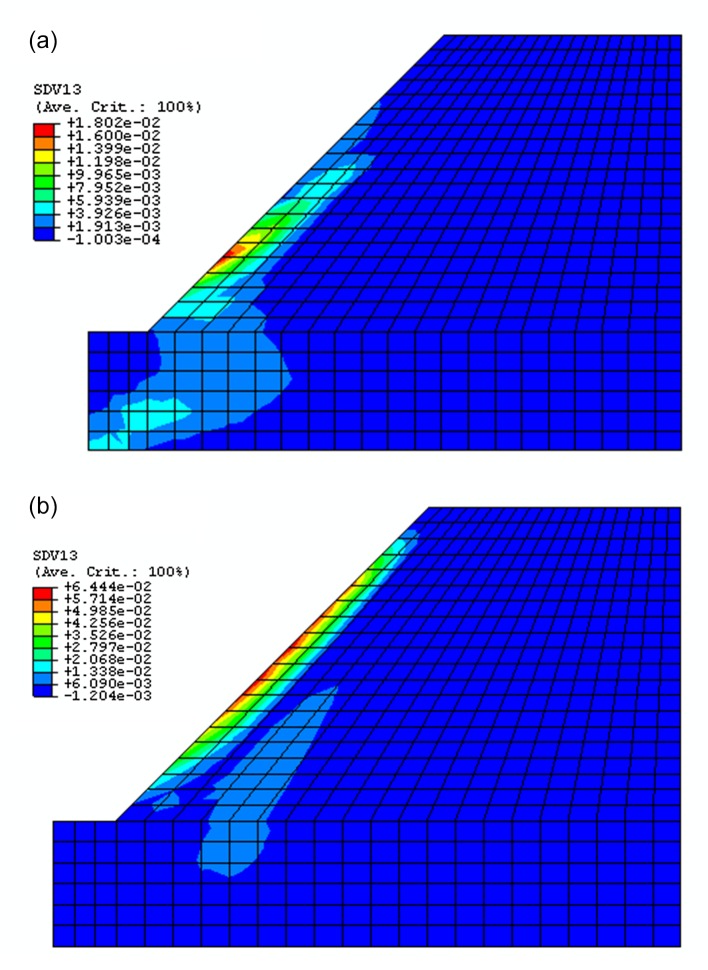
The contour for equivalent plastic deviator strain calculated by proposed constitutive model for the different slopes((a) The contour for equivalent plastic deviator strain calculated by proposed constitutive model for the first slope; (b) The contour for equivalent plastic deviator strain calculated by proposed constitutive model for the second slope)

Through observing the results of Fig 22 and Fig 23, they show that equivalent plastic strain is easy to develop along the direction, which has higher persistence ratio *k*. It is unfavorable to anti-slide stability if the visual angle *β*_0_, which has higher persistence ratio *k*, is similar to the angle of rock slope. And it shows the proposed constitutive model can consider the persistence ratio *k* in different visual angle *β*_0_.

## Conclusion

A constitutive model for jointed rock mass, which can consider the persistence ratio in different visual angle, is proposed. The proposed the yield strength criterion *f* is not only related to friction angle and cohesion of jointed rock masses at the visual angle but also related to the intersection angle between the visual angle and the directions of the maximum principal stresses.

From above analysis, it shows that the yield strength criterion *f* in proposed constitutive model is not only related to *φ*_*β*0_ and *c*_*β*0_ but also related to *β*. The yield strength criterion *f* has the relation of *k* (in other word, *φ*_*β*0_ and *c*_*β*0_) only when *β*_min_≤*β*≤*β*_max_. The relation of *β* and *β*_0_ is also important to the yield strength criterion *f*. The different relation of *β* and *β*_0_ leads to different yield strength criterion *f*. The proposed constitutive model can consider the persistence ratio *k* in different visual angle *β*_0_. The proposed constitutive model has precision to calculate displacement, stress and plastic strain. The results show the proposed constitutive model has precision to calculate displacement, stress and plastic strain.

However, the proposed constitutive model also has limitations. The model can describe the anisotropic strength of jointed rock, but cannot describe the anisotropic behaviors of elastic modulus and Poisson ratio. And the anisotropic strength of proposed model is also homogenized results. It cannot describe the localization phenomena of jointed rock precisely. And these problems need more research.

## Supporting Information

S1 File Data of Fig 5. (XLSX)

S2 File Data of Fig 6. (XLSX)

S3 File Data of Fig 8. (XLSX)

S4 File Data of Fig 12. (XLSX)

S5 File Data of Fig 21. (XLSX)
